# The DEAD-box RNA helicase 51 controls non-small cell lung cancer proliferation by regulating cell cycle progression via multiple pathways

**DOI:** 10.1038/srep26108

**Published:** 2016-05-20

**Authors:** Xiaojing Wang, Hongli Liu, Chengling Zhao, Wei Li, Huanbai Xu, Yuqing Chen

**Affiliations:** 1 Department of Respiration; Anhui Clinical and Preclinical Key Laboratory of Respiratory Diseases; First Affiliated Hospital; Bengbu Medical College; Bengbu 233000, Anhui China; 2 Department of Gynecological Oncology, First Affiliated Hospital; Bengbu Medical College; Bengbu 233000, Anhui China; 3 Department of Endocrinology and Metabolism, Shanghai Jiaotong University Affiliated First People’s Hospital, Shanghai 200080, China

## Abstract

The genetic regulation of cell cycle progression and cell proliferation plays a role in the growth of non-small cell lung cancer (NSCLC), one of the most common causes of cancer-related mortality. Although DEAD-box RNA helicases are known to play a role in cancer development, including lung cancer, the potential involvement of the novel family member DDX51 has not yet been investigated. In the current study we assessed the role of DDX51 in NSCLC using a siRNA-based approach. DDX51 siRNA-expressing cells exhibited a slower cell proliferation rate and underwent arrest in S-phase of the cell cycle compared with control cells. Microarray analyses revealed that DDX51siRNA expression resulted in the dysregulation of a number of cell signalling pathways. Moreover, injection of DDX51 siRNA into an animal model resulted in the formation of smaller tumours compared with the control group. We also assessed the expression of DDX51 in patients with NSCLC, and the data revealed that the expression was correlated with patient age but no other risk factors. Overall, our data suggest for the first time that DDX51 aids cell cancer proliferation by regulating multiple signalling pathways, and that this protein might be a therapeutic target for NSCLC.

Non-small cell lung cancer (NSCLC) is the most common type of lung cancer^1^, and was the major cause of cancer-related deaths worldwide in 2014^2^. The prognosis for lung cancer patients is generally poor and the survival rate is low^3^. Therefore, discovering the mechanisms that regulate disease pathogenesis and identifying novel potential therapeutic agents is urgent. Although environmental factors play a role in the formation of lung cancer, genetic factors also determine predisposition to the disease^4^.

The dysregulation of apoptosis is generally regarded as a genetic hallmark of cancer development^5^. Apoptosis, a tightly regulated mechanism of genetically programmed cell death, is not only involved in tumour development, but also has an active role in maintaining tissue homeostasis and controlling tumour proliferation^6^. Therefore, previous studies have identified apoptosis as a potential target for chemotherapy. At the same time, defective apoptosis might impair the effects of drug treatments and therefore result in ineffective cancer treatments^7^. Apoptosis is triggered by two distinct signalling cascades, the intrinsic and extrinsic pathways, which converge on the final apoptosis effector caspases (CASP) 3, 6, and 7^8^. The intrinsic apoptotic pathway is activated by cell damage such as apoxia and DNA damage. It is regulated by CASP9, and it is triggered by the release of cytochrome C into the cytoplasm^9^.

The DEAD-box RNA helicase (DDX) family are ubiquitously expressed proteins that are involved in RNA metabolism, including splicing, translation, pre-rRNA processing, and ribosome assembly^10^,^11^. They also play a role in regulating the intrinsic apoptotic pathway. For example, DDX51 is a negative regulator of the apoptotic effector p53^11^, and thereby actively promotes cell proliferation^12^. Moreover, DDX5 expression is dysregulated in different types of cancers^13^,^14^,^15^, including NSCLC. Specifically, DDX5 might promote cell proliferation in NSCLC by activating the transcription of cyclin D1 to promote cell cycle progression^12^. Although DDX5 has a role in promoting cell proliferation in NSCLC, the roles of other members of the family are more elusive. For example, DDX51 is involved in regulating RNA metabolism, and in particular in the maturation of pre-RNAs^10^. However, the clinical importance of this protein in the context of NSCLC has not been assessed previously.

In the current study we used a siRNA silencing approach to investigate the role of DDX51 as a transcriptional regulator in NSCLC for the first time. DDX51siRNA H1299 cell cultures exhibited a slower proliferation rate, underwent cell cycle arrest in S phase, and displayed a higher percentage of apoptotic cells. Moreover, microarray analyses showed a change in the expression of signalling-related genes in these cells, suggesting that the cell proliferation defects in DDX51siRNA H1299 cells might be linked to a change in transcriptional regulation. DDX51siRNA H1299 xenografts in mice formed smaller tumours compared with control cells, suggesting that the protein also has a role *in vivo*. Taken together, these data suggest that DDX51 regulates cell proliferation by facilitating the transition between the S and G2 phases of the cell cycle, likely by affecting the transcriptome of different signalling pathways. These results also suggest that DDX51 plays an important role clinically, indicating that it is a potential target for future therapeutic applications.

## Materials and Methods

### Cell lines

H1299 cells (American Type Culture Collection, USA) were cultured in DMEM (Gibco, USA) supplemented with 10% foetal calf serum (Thermo Fisher Scientific, USA), 100 μg/ml streptomycin (Gibco, USA), and 100 U/ml penicillin (Gibco, USA) at 37 °C with 5% CO_2_.

### siRNA cloning and lentiviral transfection of H1299 cells

The lentiviral vectors were purchased from Shanghai Genechem Company Ltd., China. A non-silencing siRNA (5′-GCCTAACTGTGTCAGAAGGAA-3′) was used as the negative control (NC). The siRNA sequences targeting DDX51 gene were 5′-CCTATTTCCCATGATTCCTTCATA-3′ and 5′-GTAATACGGTTATCCACGCG-3′. The cells were seeded into a six-well plate (~5 × 10^4^ cells per well) and incubated at 37 °C with 5% CO_2_ until they reached ~30% confluence before transfection.

### Cancer patient samples

Material was harvested from 75 fresh matched pairs of tissues samples isolated from lung cancer patients who were hospitalized in the affiliated hospital of Bengbu Medical College (Bengbu, China) between March and July 2013. Written informed consent was obtained from all patients. The Ethics Committee of the Affiliated Hospital of Bengbu Medical College approved the study protocol, and all experiments were performed in accordance with the approved guidelines.

### Real-time quantitative PCR (RT-qPCR)

RNA extraction was performed using TRIzol (Invitrogen, USA) according to the manufacturer’s instructions. cDNA was synthetized by reverse transcription using an ABI 2720 thermal cycler (ABI Biosystems, USA) according to the manufacturer’s instructions (M-MLV-RTase, Promega, USA). The cDNA product was detected using a SYBR Green Supermix kit (Toyobo, Osaka, Japan) with a Takara Bio PCR Thermal Cycler Dice Real Time TP800 (Takara, Japan). The cycling parameters were 95 °C for a 30-s hot start followed by 45 cycles of 95 °C for 5 s and 60 °C for 30 s. The relative mRNA expression (EIF3B/GAPDH) was determined using the 2^−ΔCt^ method. The sequences of the primers used are presented in Supporting Table SX.

### Lung cancer tissue microarrays

Lung cancer tissue microarrays (TMAs) were constructed at Shanghai Outdo Biotech Co., Ltd (Shanghai, China). Rabbit polyclonal anti-human DDX51 antibodies (1:150) were used for immunohistochemistry according to a two-step protocol. The protein expression patterns of DDX51 were analysed in 75 lung cancer tissues and paired adjacent noncancerous tissues. The staining intensity score was assigned as follows: 0 points, negative; 1 point, low; 2 points, medium; and 3 points, high. The staining percentage score was as follows: 0% positive cells, 0; 1–25% positive cells, 1; 26–50% positive cells, 2; 51–75% positive cells, 3; and ≥76% positive cells, 4. The “DDX51 final score” was calculated as the combination of the “staining intensity score” and the “staining percentage score”. Patients were grouped into “low expression” (total score of 0–5) and “high expression” (total score of 6–12) groups according to their :DDX51 scores”. The difference in DDX51 expression between tumour and non-tumour tissue was determined using paired Wilcoxon tests. The association between clinicopathological parameters and DDX51 expression was analysed using chi-square tests. The association between DDX51 expression and overall survival was evaluated using Mantel-Cox log-rank tests. Overall survival time was defined as the number of months from the date of histological diagnosis to the date of last contact or death from any cause. Patients who were alive at the last follow-up or were lost to follow-up were censored. Survival curves were plotted using the Kaplan-Meier method.

### Cell proliferation assays

After achieving the logarithmic growth phase H1299 cells were trypsin-digested, resuspended in standard medium, and then seeded into 96-well plates at a density of 2,000 cells/well. The number of GFP fluorescence-positive cells was counted using a Cellomics Array Scan High Contents Screening Reader on five consecutive days.

### Apoptosis assays

Apoptosis was assessed using annexin V-based flow cytometry using standard laboratory methodology. Briefly, cells were transfected as described above, and incubated for 5 days. The cells were then harvested, resuspended in binding buffer at a density of 1 × 10^6^ cells/ml, and 100 μl of this suspension was added to FACS tubes and stained with annexin V. Cells were mixed gently in a dark room for 15 min at room temperature, and then analysed using flow cytometry. Although standard flow cytometry apoptosis assays use co-staining with propidium iodide, we analysed only annexin V since the cells were transfected with a GFP-expressing vector and the excitation wavelengths of propidium iodide and GFP overlap.

### Colony formation assays

After reaching the logarithmic growth phase H1299 cells were trypsinized, counted, and seeded at a density of 800 cells/well into six-well plates containing regular culture medium. After 14 days the cells were washed twice with PBS, fixed with methanol, and stained with Giemsa. The colonies were then photographed and scored.

### Cell cycle assay

Lentivirus-transfected cells were cultured in 6-cm dishes until they reached 80% confluence, and were then trypsinized, washed twice in PBS, and fixed with 70% pre-chilled ethanol at 4 °C for 1 h. The fixed cells were washed and stained with a propidium iodide (PI) mixture containing 50 μg/ml PI and 100 μg/ml ribonuclease in PBS for 45 min at 37 °C. The cells were passed through a 300-mesh nylon net, before the DNA content was determined using quantitative flow cytometry with the standard optics of a FACScan flow cytometer (Becton–Dickinson FACS Calibur). All experiments were performed in triplicate.

### Tumour growth in nude mice

H1299 cells (5 × 10^6^) were suspended in 100 μL serum-free Dulbecco’s modified Eagle medium and Matrigel (BD Biosciences, San Jose, CA; 1:1) and implanted subcutaneously into the flanks of BALB/c nu/nu male nude mice (10 mice/group, 4–6 weeks old; Institute of Materia Medica, Chinese Academy of Sciences, Shanghai, China). All mice were monitored once every 3 days and were sacrificed after 5 weeks. All procedures were approved by the animal care and use committee of the Affiliated Hospital of Bengbu Medical College, and all experiments were performed in accordance with the approved guidelines.

### Microarray gene expression analysis

After transfection with DDX51-siRNA total RNA was extracted from H1299 cells, and 50–500 ng of RNA was used to generate biotin-modified amplified RNA (aRNA) using a GeneChip 3′ IVT Express Kit (Affymetrix, USA). Reverse transcription was performed using a T7 oligo (dT) primer and a first-strand IVT Labelling Master Mix was used to produce multiple copies of biotin-modified aRNA. The aRNA was then purified and quantified. After fragmentation the aRNA was hybridized to the GeneChip Human Genome U133 Plus 2.0 Array (Affymetrix, USA). After hybridization the chips were stained with phycoerythrin and washed in a Genechip Fluidics Station 450. The microarray signals were scanned and analysed using a Genechip Array Scanner 3000 7G.

### Bioinformatics

Differentially expressed genes (DEGs) between the DDX51-siRNA and NC groups with corrected p-values of <0.05 and a fold change of >2.0 or <0.5 were considered to be significantly differentially expressed. Gene Ontology (GO) enrichment analysis and Kyoto Encyclopaedia of Genes and Genomes and BioCarta pathway databases were performed on significant DEGs.

### Western blotting

The treated cells were harvested and proteins were extracted using a protein extraction kit (Beyotime, China). Proteins were separated using SDS–PAGE and then transferred to PVDF membranes at 4 °C (300 mA for 150 min). Membranes were blocked in 5% skim milk in TBST overnight at 4 °C, and then incubated with primary antibodies overnight at 4 °C. They were then incubated with secondary antibodies. After washing three times in TBST the membranes were incubated in Amersham™ ECL Plus Western Blotting Detection System (GE Healthcare, UK) before visualization.

### Statistical analysis

Statistical analyses were performed using GraphPad Prism and GraphPad InStat software (GraphPad Software, La Jolla, CA, USA). *T*-tests were used to determine significance, with a *p*-value of <0.05 to indicate a significant difference.

## Results

**Figure 1:**
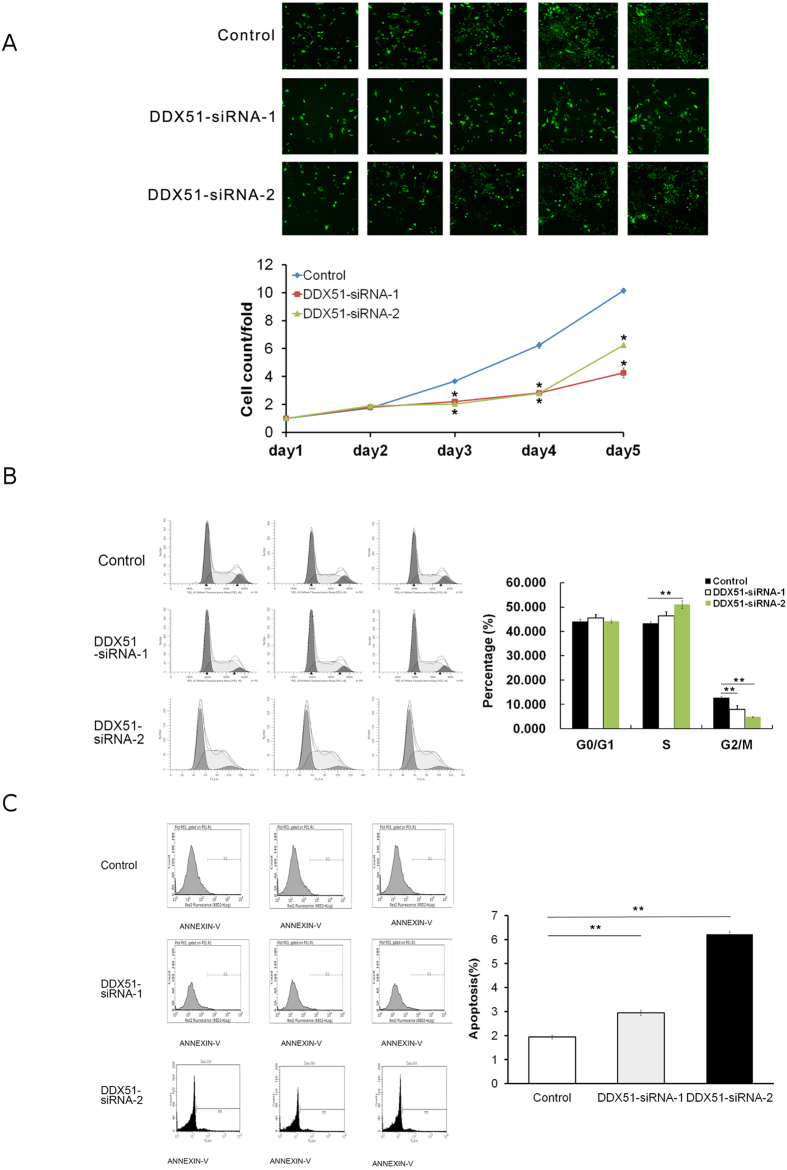
Effects of knocking down DDX51 on cell proliferation, the cell cycle, and apoptosis. (**A**) Cellomic assay pictures and fluorescence quantification over 5 days after transfection in mock transfected and two DDX51-siRNA H1299 cell lines. (**B**) Cell cycle analysis using FACS in mock transfected and two DDX51-siRNA H1299 cell lines. Graphs illustrate representative examples. Bar graphs indicate the mean percentages. (**C**) Apoptosis was analysed in mock transfected and two DDX51-siRNA H1299 cell lines using FACS after staining with annexin V. Graphs show representative examples and report the values of three biological replicates. Bar graphs indicate the mean percentage values. Error bars, standard error. *P < 0.01.

In order to fully understand the role of DDX51 in NSCLS we used a siRNA approach to target the transcription of DDX51. We first assessed the effectiveness of our siRNA in the human NSCLC cell line H1299 (Supplementary Fig. 1), and then measured the proliferation rate of DDX51-siRNA lines. The Cellomics assay revealed that DDX51siRNA cells had a significantly slower proliferation rate than control cells after 3, 4, and 5 days (*P* < 0.01; Fig. 1A). We investigated this slow growth phenotype further by analysing the FACS profile of DDX51-siRNA cell cultures. The results indicated that there were a higher percentage of DDX51-siRNA H1299 cells in S phase compared with the control cells (*P* < 0.01). Conversely, there were fewer DDX51-siRNA than control H1299 cells in G2/M2 phase (*P* < 0.01; Fig. 1B). Overall, these data suggest that the DDX51 siRNA construct inhibited cell proliferation by blocking cell cycle progression in S phase.

Because cell cycle arrest coincided with apoptosis, we next investigated whether the expression of DDX51 siRNA induced apoptosis in H1299 cells. FACS analysis of Annexin V-stained cells demonstrated that the percentage of DDX51-siRNA H1299 cells undergoing apoptosis was significantly higher (*P* < 0.01) compared with the control group (Fig. 1C).

**Figure 2:**
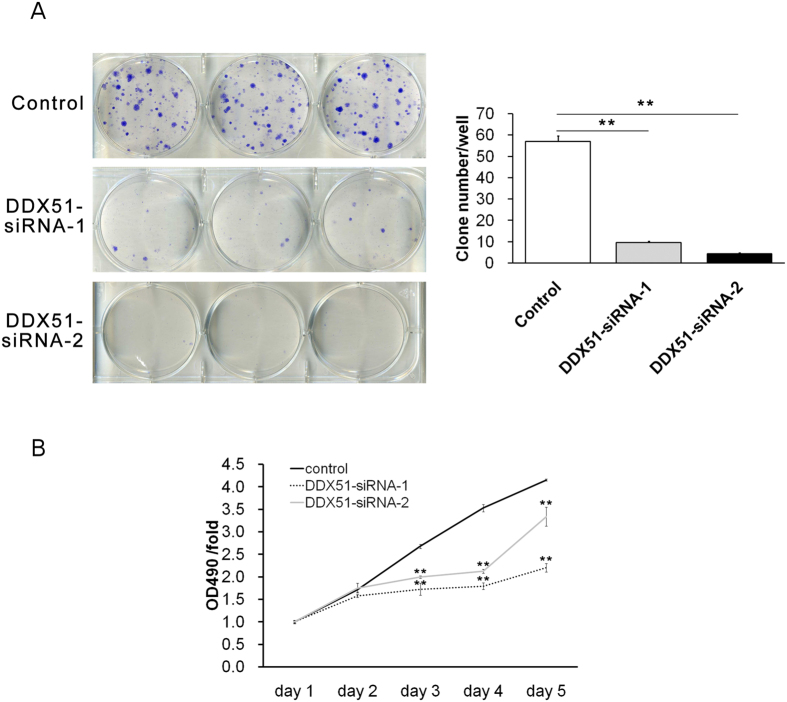
Effects of knocking down DDX51 on colony formation and cell proliferation in H1299 cells. (**A**) Sample images Giemsa-stained mock transfected and DDX51 siRNA-expressing H1299 cells with quantification. (**B**) MTT assays were performed in mock transfected and DDX51 siRNA-expressing H1299 cells. Graphs show the mean percentages of three biological replicates. Error bars, standard error. *P < 0.01.

Finally, colony formation assays demonstrated that the expression of DDX51 siRNA dramatically reduced the ability of H1299 cells to form colonies (Fig. 2), consistent with our cell proliferation and apoptosis observations.

Because DDX51 plays a role in regulating RNA processing (10), it is possible that its effects on the cell cycle and apoptosis could be elicited by changes in the transcriptome of H1299 cells. Therefore, we profiled the gene expression of DDX51-siRNA H1299 cells using microarrays. Our analysis showed that H1299 DDX51-siRNA cells had significant changes in the transcription of several signalling-related genes compared with negative control cells (Supplementary Fig. 2). The results demonstrated that 122 and 137 genes were up- and down-regulated in DDX51 knockout cells, respectively. Genes upregulated significantly included *RAPA1, MAP4K4, IL1R1, JUN, FOS, TGFBR2*, and *HSPA8*. The genes downregulated significantly in the presence of reduced levels of DDX51 included *MAP2K5* and *MKNK2* (Supplementary Table 1). The microarray results were also assessed using GO analyses, and the results are shown in Supplementary Fig. 3 and Supplementary Table 2.

**Figure 3:**
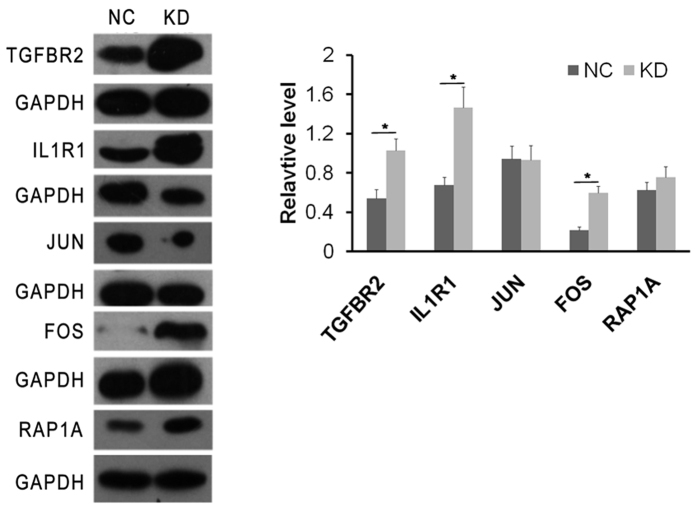
The effects of DDX51 knockdown on protein expression according to western blotting. GAPDH was used as a loading control. NC, negative control; KD, DDX51 knockdown. All results were reproducible in three independent experiments. *P < 0.05.

The microarray results were validated by analysing the expression of a group of representative proteins using western blotting. Data revealed that the expression of TGF-βR1, IL1-R1, and C-FOS was increased in cells expressing DDX51 siRNA (Fig. 3), confirming the microarray data. These data suggest that the effects of DDX51 siRNA on the cell cycle and apoptosis were likely dependent on the broader regulation of cellular functions.

**Figure 4:**
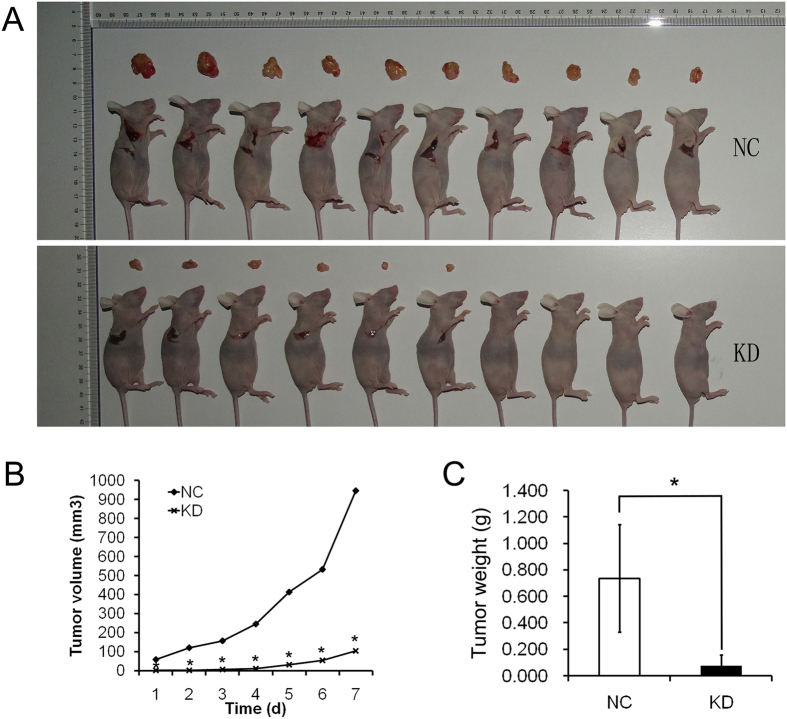
Effects of DDX51 knockdown on xenograft tumorigenicity *in vivo* in nude mice. H1299/NC and H1299/KD cells were injected into the flanks of nude mice. Tumour growth (**A**) and tumour volume (**B**) were measured on the indicated days. Tumour weights were measured 4 weeks after injection (**C**). NC, negative control; KD, DDX51 knockdown. *P < 0.05.

To confirm the *in vitro* results we next investigated the effects of suppressing DDX51 expression in an animal model. Wild type and DDX51 siRNA-expressing H1299 cells were implanted into nude BALB/c nu/nu mice, and tumour progression was monitored for 1 week before the animals were sacrificed. The volume of DDX51-siRNA H1299 tumours was significantly smaller than control tumours throughout the experiment (Fig. 4A,B). Similarly, DDX51-siRNA H1299 tumours were lighter at the end of the observation period compared with control tumours (Fig. 4C). These results suggest that DDX51 plays a role in cancer growth *in vivo*.

**Figure 5:**
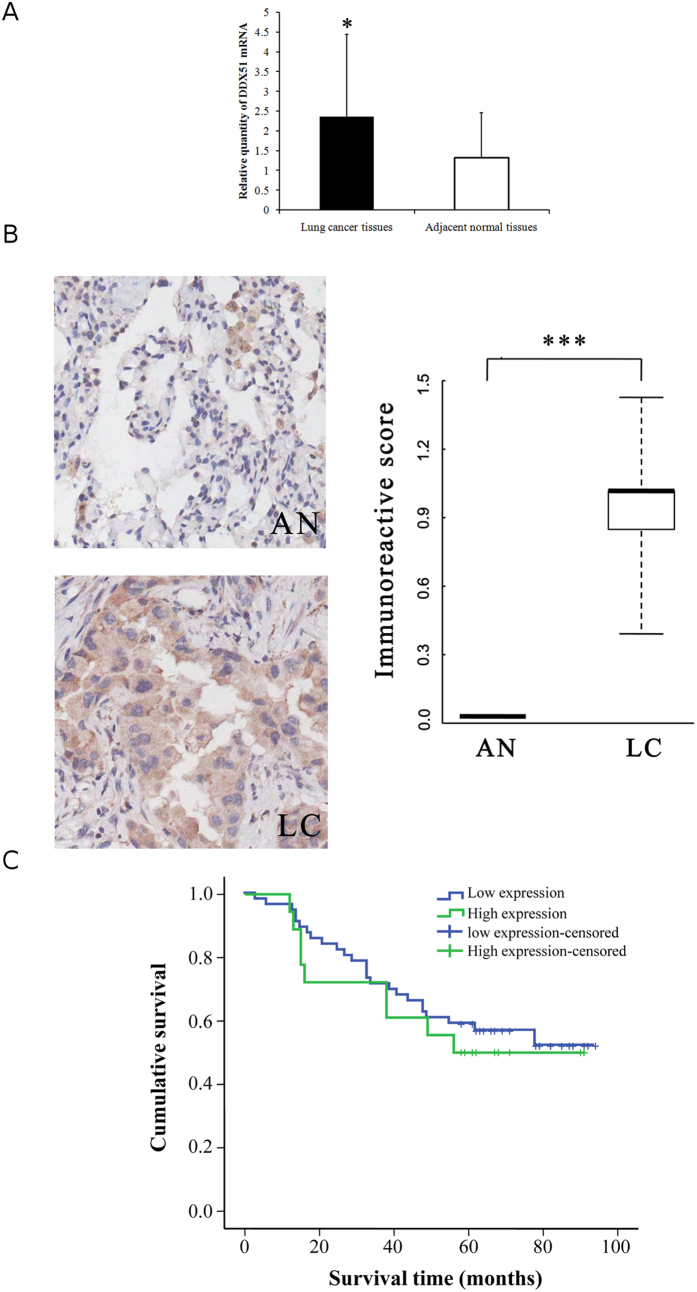
Analysis of DDX51 expression and the correlation between DDX51 expression and survival in cancer patients. (**A**) RT-qPCR analysis of DDX51 expression in lung cancer and adjacent normal tissues. The relative levels of DDX51 mRNA in lung cancer tissues was normalized to GAPDH and compared with the expression in adjacent normal tissues. Graphs show the mean values of three biological repeats. (**B**) Immunohistochemical analysis of DDX51 expression in lung cancer and adjacent normal tissues. Semi-quantitative analysis of the stained sections was performed using light microscopy to calculate the immunoreactive score (see methods). AN, adjacent normal tissues; LC, lung cancer tissues. Kaplan–Meier curve showing the relationship between DDX51 expression and the survival of lung cancer patients. Graphs show the mean percentage values. Error bars, standard error. *P < 0.01.

To confirm this hypothesis and elucidate the mechanism of action of DDX51 in NSCLC *in vivo* we analysed tissues that had been harvested from hospitalized NSCLC patients (Table 1). DDX51 was overexpressed in cancer tissues compared with neighbouring regions (*P* < 0.05; paired sample test) according to both qRT-PCR and immunohistochemistry (Fig. 5A,B). Therefore, we investigated the relationship between DDX51 expression and clinicopathological factors in NSCLC patients (Table 1). There were no differences in any factors between patients with high or low levels of DDX51 expression (Table 1). Similarly, there was no difference in the survival of patients in the two groups (Fig. 5C). No other lung cancer risk factors, such as N or TNM stage, had a high correlation with DDX51 expression (Table 2).

## Discussion

The current study demonstrated that DDX51 facilitates cell cycle progression and therefore promotes cell proliferation. Since the DDX51-siRNA cells were arrested in S-phase, we propose that DDX51 regulates the progression through S phase. The current results also suggest that this is the result of regulating a number of different signalling pathways, as indicated by the microarray data. This hypothesis is consistent with a role for DDX51 in ribosome assembly and RNA metabolism^10^, as well as with previous reports revealed a function for other DDX family members in regulating the cell cycle via different signalling pathways^11^,^12^,^13^.

The current microarray and western blot data demonstrated that DDX51 siRNA-expressing cells expressed higher levels of TGF-β-R, IL1R, and C-FOS. TGFβ has a dual role in lung cancer, since it both promotes and inhibits cell proliferation^16^. Some studies have suggested that the role of TGF-β might depend on its concentration. Although some studies have suggested that low concentrations were correlated with a role in tumour suppression^17^,^18^, knocking down DDX51 in the current study increased TGFBR2 levels, slowed cell proliferation, and delayed cancer progression. It is also difficult to explain the overexpression of C-FOS in DDX51-siRNA cells since it is generally regarded as an oncogene^19^. Therefore, future studies are needed to address these apparently contradictory data. Although they were not studied functionally in the current study, other genes whose expression was altered in the DDX51 might also be important in NSCLC and warrant further investigation in future studies. For example, the expression of three MAPK-related genes was altered (MAP4K4, MAP2K5, and MKNK2), and this pathway plays an important role in the survival of a number of different tumour cells^20^,^21^. In addition, HSPA8 polymorphisms have been reported in a number of different types of cancer^22^,^23^.

The results of xenografts experiments confirmed that depleting DDX51 expression slowed cancer progression in an animal model. Although the current experiment did not analyse the effects of silencing DDX51 on mouse mortality, the reduced sized tumours formed by DDX51-silenced cells suggests that DDX51 might be a good target for cancer therapy. Consistent with this, our data suggested that DDX51 expression was significantly higher in cancer tissues compared with non-tumour control tissues. However, it is currently unclear if this is an indication of the severity of the cancer itself. While the current data indicate that inhibiting DDX51 expression reduced the proliferation of tumours, future studies will have to address whether the dysregulated expression of DDX51 alone is sufficient to induce uncontrolled cell replication. Because DDX51 expression correlated with cell proliferation and hence the cell cycle, these data are also consistent with the known role of DDX51 in ribosome processing^10^, as well as potentially protein translation during replication.

In conclusion, the current results confirmed the importance of the DDX family and pre-rRNA processing in the proliferation of cancer cells, and revealed a role for DDX51 in this process for the first time. Moreover, these data provide an important starting point for future studies investigating the potential of DDX51 as a target for pharmacological screens for the development of new potential anti-cancer drugs.

## Additional Information

**How to cite this article**: Wang, X. *et al*. The DEAD-box RNA helicase 51 controls non-small cell lung cancer proliferation by regulating cell cycle progression via multiple pathways. *Sci. Rep.* **6**, 26108; doi: 10.1038/srep26108 (2016).

## Supplementary Material

Supplementary Information

## Figures and Tables

**Table 1:** The relationship between DDX51 expression and clinicopathological factors in lung cancer.

**Characteristics**	**DDX51 expression**		***P*** **value**
	**Low**	**High**	
Age (years)			
≤60	35	7	0.14
>60	22	10	
Loss	0	1	
Tumour size			
≤3.5 cm	29	10	0.729
>3.5 cm	28	8	
Loss	0	0	
Gender			
Male	31	8	0.462
Female	26	10	
Loss	0	0	
Pathological grade			
I	7	3	0.139
II	29	13	
III	20	2	
Loss	1	0	
T stage			
T1, T2	47	16	0.516
T3, T4	10	2	
Loss	0	0	
N stage			
Nx, N0	38	13	0.66
N1, N2, N3	19	5	
Loss	0	0	
M stage			
M0	53	18	0.316
M1	3	0	
Loss	1	0	
TNM stage			
TNM1, TNM2	36	10	0.951
TNM3, TNM4	15	4	
Loss	6	4	

**Table 2:** Spearman correlation analysis between DDX51 expression level and clinical pathologic factors.

**Variable**	**Gender**	**Age**	**Tumour size**	**Pathological grade**	**T stage**	**N stage**	**M stage**	**TNM stage**
Case number	75	74	75	74	75	75	74	65
Correlation Coefficient	0.090	0.128	0.040	−0.061	−0.024	−0.034	−0.072	0.067
*P* value	0.444	0.277	0.736	0.606	0.836	0.773	0.541	0.597
