# Supplementary material for: The DEAD-box RNA helicase 51 controls non-small cell lung cancer proliferation by regulating cell cycle progression via multiple pathways
Source: Sci Rep. 2016 May 20;6:26108. doi: 10.1038/srep26108 (PMC4873746; doi:10.1038/srep26108)
Supplement: Supplementary Information [file srep26108-s1.pdf]

Supplementary Information – title page

The DEAD-box RNA helicase 51 controls non-small cell lung cancer proliferation by regulating cell cycle progression via multiple pathways

Xiaojing Wang[1], Hongli Liu[2], Chengling Zhao[1], Wei Li[1], Huanbai Xu[3], and Yuqing Chen[1]*

[1]Department of Respiration; Anhui Clinical and Preclinical Key Laboratory of Respiratory Diseases; First Affiliated Hospital; Bengbu Medical College; Bengbu 233000, Anhui China;

[2]Department of Gynecological Oncology, First Affiliated Hospital; Bengbu Medical College; Bengbu 233000, Anhui China;

[3]Department of Endocrinology and Metabolism, Shanghai Jiaotong University Affiliated First People's Hospital, Shanghai 200080, China

Running title: DDX51 regulates cell cycle progression in NSCLC

Gene Symbol	Regulation	Fold Change
RAP1A	up	2.5165434
MAP4K4	up	2.1900065
DDX51	down	2.1816182
IL1R1	up	2.0928142
JUN	up	2.0195925
FOS	up	1.7622768
MAP2K5	down	1.7612897
TGFBR2	up	1.6535834
MKNK2	down	1.5812604
HSPA8	up	1.520771

Supplementary Table 1. Genes up- and down-regulated in DDX51 knockdown cells

Supplementary Table 2. The results of gene ontology analysis in DDX51 knockdown cells.

**GO analysis results (biological processes)**			
Gene Set Name	Gene number	significance probability	Gene Name
POSITIVE_REGULATION_OF_CELLULAR_PROCESCESS	18	2.16E-05	FOSL1, CITED2, BMP6, NAA15, ACVR2B, BCLAF1, EGR1, PTEN, SCG2, VEGFA, ASNS, CDKN2B, LIF, SSR1, CLEC11A, TGFBR2, BBC3, TGM2
POSITIVE_REGULATION_OF_BIOLOGICAL_PROCROCESS	18	2.64E-05	FOSL1, CITED2, BMP6, NAA15, ACVR2B, BCLAF1, EGR1, PTEN, SCG2, VEGFA, ASNS, CDKN2B, LIF, SSR1, CLEC11A, TGFBR2, BBC3, TGM2
REGULATION_OF_NUCLEOBASENUCLEOSIDENUCLUCLEOTIDE_AND_NUCLEIC_ACID_METABOLIC_PC_PROCESS	16	5.91E-05	FOSL1, CITED2, BMP6, NAA15, ACVR2B, BCLAF1, EGR1, FOS, KLF10, TCFL5, HEXIM1, PBX3, KLF7, SRSF10, PAIP1, ATP8B1
NUCLEOBASENUCLEOSIDENUCLEOTIDE_AND_NUCNUCLEIC_ACID_METABOLIC_PROCESS	23	5.91E-05	FOSL1, CITED2, BMP6, NAA15, ACVR2B, BCLAF1, EGR1, FOS, KLF10, TCFL5, HEXIM1, PBX3, KLF7, SRSF10, PAIP1, ATP8B1, ATM, RAD23B, CELF1, PRPF31, RBMS2, NT5E, MTAP
BIOPOLYMER_METABOLIC_PROCESS	27	6.96E-05	FOSL1, CITED2, BMP6, NAA15, PTEN, FOS, KLF10, TCFL5, HEXIM1, PBX3, KLF7, SRSF10, PAIP1, ATM, RAD23B, CELF1, PRPF31, RBMS2, NT5E, PLAT, MAP4K4, SERP1, MKNK2, SSH1, ALG5, TWF1, KDM4A
RESPONSE_TO_STRESS	14	1.01E-04	SCG2, VEGFA, ASNS, FOS, ATM, RAD23B, PLAT, MAP4K4, SERP1, CTGF, TFPI, DNAJB1, HERPUD1, SRXN1
REGULATION_OF_CELL_PROLIFERATION	11	1.15E-04	FOSL1, PTEN, SCG2, VEGFA, CDKN2B, LIF,

			SSR1, CLEC11A, TGFBR2, KLF10, TCFL5
REGULATION_OF_CELLULAR_METABOLIC_PROCEOCESS	17	1.43E-04	FOSL1, CITED2, BMP6, NAA15, ACVR2B, BCLAF1, EGR1, CDKN2B, FOS, KLF10, TCFL5, HEXIM1, PBX3, KLF7, SRSF10, PAIP1, ATP8B1
REGULATION_OF_METABOLIC_PROCESS	17	1.56E-04	FOSL1, CITED2, BMP6, NAA15, ACVR2B, BCLAF1, EGR1, CDKN2B, FOS, KLF10, TCFL5, HEXIM1, PBX3, KLF7, SRSF10, PAIP1, ATP8B1
REGULATION_OF_TRANSCRIPTION	14	2.14E-04	FOSL1, CITED2, BMP6, NAA15, ACVR2B, BCLAF1, EGR1, FOS, KLF10, TCFL5, HEXIM1, PBX3, KLF7, ATP8B1

GO analysis results (cellular components)			
Gene Set Name	Gene number	significance probability	Gene Name
CYTOPLASM	39	4.23E-09	SMC2, NAA15, CDKN2B, HEXIM1, SEPT2, MTMR2, SLC17A5, TFRC, GOLM1, SSR1, NECAP2, SEC22A, HERPUD1, UQCRB, ACVR2B, HCCS, PEX19, SSH1, TUBE1, SERP1, PGAP1, TMEM117, C10orf10, BBC3, PC, PCK2, EIF2S2, SRXN1, YWHAB, GARS, CARS, KRT18, MYO5A, PAIP1, RCHY1, DDIT4, PTEN, SESN2, PDXK
NUCLEUS	24	8.58E-05	SMC2, NAA15, CDKN2B, HEXIM1, SEPT2, MTMR2, PRPF31, JUN, SRSF10, CITED2, TCFL5, EGR1, FHL2, FOS, GNL2, NR4A2, PBX3, TRIB3, RAD23B, KLF10, WEE1, WRB, FOSL1, KDM4A

MEMBRANE	29	8.58E-05	SLC17A5, TFRC, GOLM1, SSR1, NECAP2, SEC22A, HERPUD1, UQCRB, ACVR2B, HCCS, PEX19, SSH1, SLC1A4, ITGA10, COL17A1, GPC6, EFNB2, IL1R1, LIFR, CD200, AXL, SDC1, SLC7A1, CD68, TGFBR2, C7, SLC7A11, ATP8B1, CTGF
MEMBRANE_PART	25	2.27E-04	SLC17A5, TFRC, GOLM1, SSR1, NECAP2, SEC22A, HERPUD1, UQCRB, ACVR2B, SLC1A4, ITGA10, COL17A1, GPC6, EFNB2, IL1R1, LIFR, CD200, AXL, SDC1, SLC7A1, CD68, TGFBR2, C7, SLC7A11, ATP8B1
CYTOPLASMIC_PART	21	9.05E-04	SLC17A5, TFRC, GOLM1, SSR1, NECAP2, SEC22A, HERPUD1, UQCRB, HCCS, PEX19, TUBE1, SERP1, PGAP1, TMEM117, C10orf10, BBC3, PC, PCK2, EIF2S2, SRXN1, YWHAB
INTEGRAL_TO_MEMBRANE	20	1.37E-03	SLC17A5, TFRC, GOLM1, SSR1, ACVR2B, SLC1A4, ITGA10, COL17A1, GPC6, EFNB2, IL1R1, LIFR, CD200, AXL, SDC1, SLC7A1, CD68, TGFBR2, C7, SLC7A11
INTRINSIC_TO_MEMBRANE	20	1.41E-03	SLC17A5, TFRC, GOLM1, SSR1, ACVR2B, SLC1A4, ITGA10, COL17A1, GPC6, EFNB2, IL1R1, LIFR, CD200, AXL, SDC1, SLC7A1, CD68, TGFBR2, C7, SLC7A11
PLASMA_MEMBRANE_PART	17	5.30E-03	SLC17A5, TFRC,

			GOLM1, NECAP2, ACVR2B, SLC1A4, ITGA10, COL17A1, GPC6, EFNB2, IL1R1, LIFR, CD200, AXL, SDC1, SLC7A1, ATP8B1
PLASMA_MEMBRANE	19	6.34E-03	SLC17A5, TFRC, GOLM1, NECAP2, ACVR2B, SSH1, SLC1A4, ITGA10, COL17A1, GPC6, EFNB2, IL1R1, LIFR, CD200, AXL, SDC1, SLC7A1, ATP8B1, CTGF
INTEGRAL_TO_PLASMA_MEMBRANE	15	6.34E-03	SLC17A5, TFRC, GOLM1, ACVR2B, SLC1A4, ITGA10, COL17A1, GPC6, EFNB2, IL1R1, LIFR, CD200, AXL, SDC1, SLC7A1

**GO analysis results (molecular function)**			
Gene Set Name	Gene number	significance probability	Gene Name
ACTIVE_TRANSMEMBRANE_TRANSPORTER_ACTIVTIVITY	7	1.77E-03	SLC7A11, SLC7A5, SLC1A4, SLC3A2, SLC17A5, SLC6A8, ATP8B1
PHOSPHOTRANSFERASE_ACTIVITY_ALCOHOL_GR_GROUP_AS_ACCEPTOR	10	2.82E-03	ACVR2B, TGFBR2, AXL, MKNK2, MAP4K4, ATM, TWF1, PFKFB2, PFKP, PDXK
NEUTRAL_AMINO_ACID_TRANSMEMBRANE_TRANSANSPORTER_ACTIVITY	3	3.29E-03	SLC7A11, SLC7A5, SLC1A4
KINASE_ACTIVITY	10	3.29E-03	ACVR2B, TGFBR2, AXL, MKNK2, MAP4K4, ATM, TWF1, PFKFB2, PFKP, PDXK
TRANSFERASE_ACTIVITY_TRANSFERRING_PHOSHOSPHORUS_CONTAINING_GROUPS	10	7.75E-03	ACVR2B, TGFBR2, AXL, MKNK2, MAP4K4, ATM, TWF1, PFKFB2, PFKP, PDXK
SECONDARY_ACTIVE_TRANSMEMBRANE_TRANSPOSPORTER_ACTIVITY	4	7.75E-03	SLC7A11, SLC3A2, SLC17A5, SLC6A8
AMINO_ACID_TRANSMEMBRANE_	3	2.55E-02	SLC7A11, SLC7A5,

TRANSPORTER_AR_ACTIVITY			SLC1A4
RNA_BINDING	7	2.55E-02	EIF2S2, PAIP1, HEXIM1, SRSF10, CELF1, RBMS2, CARS
GROWTH_FACTOR_BINDING	3	2.87E-02	ACVR2B, IL1R1, CTGF
HEMATOPOIETIN_INTERFERON_CLASSD200_DOMDOMAIN_CYTOKINE_RECEPTOR_ACTIVITY	3	2.87E-02	IL1R1, IL13RA2, LIFR

Supplementary Figure Legends

Supplementary Figure 1. Validation of DDX51 knockdown efficiency using RT-qPCR. RT-qPCR was used to quantify DDX51 in mock transfected and two knockout H1299 cell lines. Error bars represent means ± SD. **, $P<0.01$.

Supplementary Figure 2. (A) Cluster analysis of the mRNAs differentially expressed in DDX51 knockdown and control cells (n=3). (B) Significantly changed signal pathways according to Pathway Analysis for differentially expressed genes.

Supplementary Figure 3. Gene ontology analysis of significant differentially expressed genes regulated by DDX51. DDX51-regulated genes that were differentially expressed were analyzed in the following categories: biological processes (A), cellular components (B), and molecular functions (C). The top 10 categories are shown according to –log10 (P value). Significance refers to the –log10 (P value), which was obtained using the Ingenuity program with right-tailed Fisher's exact test.

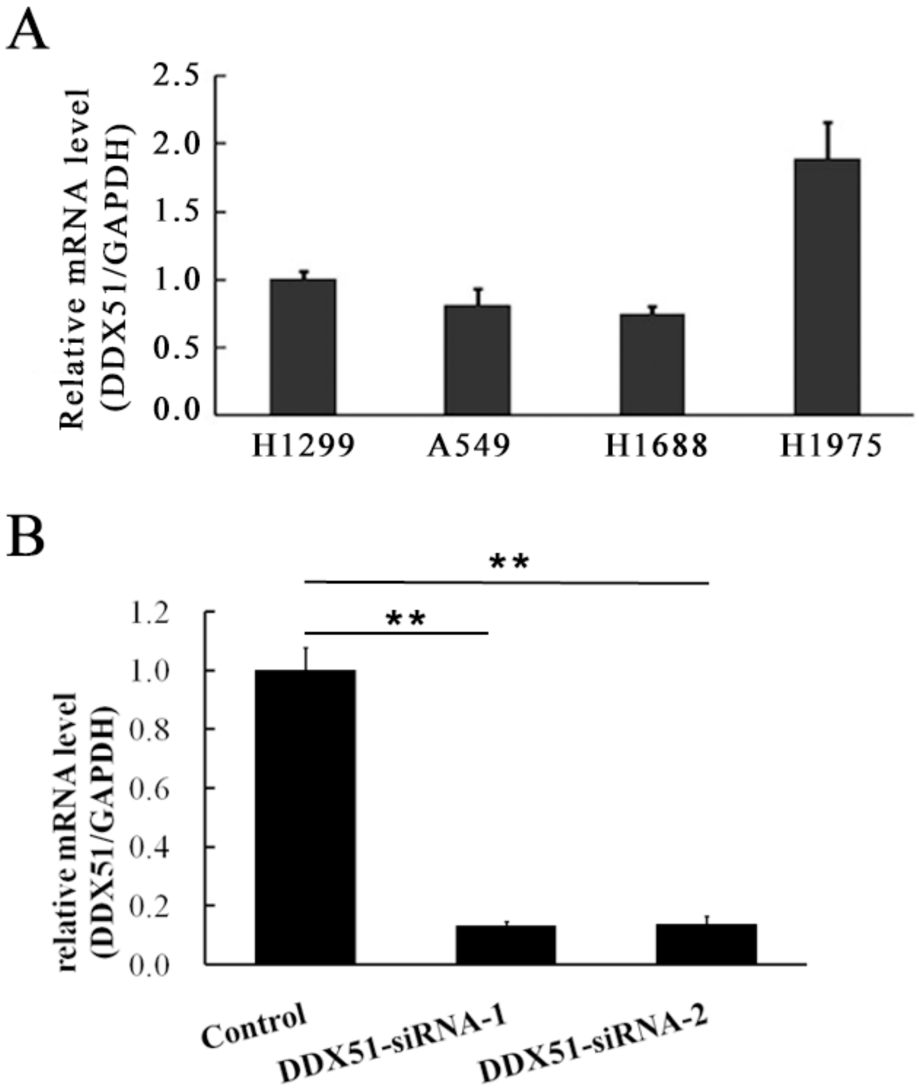

Supplementary Figure 1

A

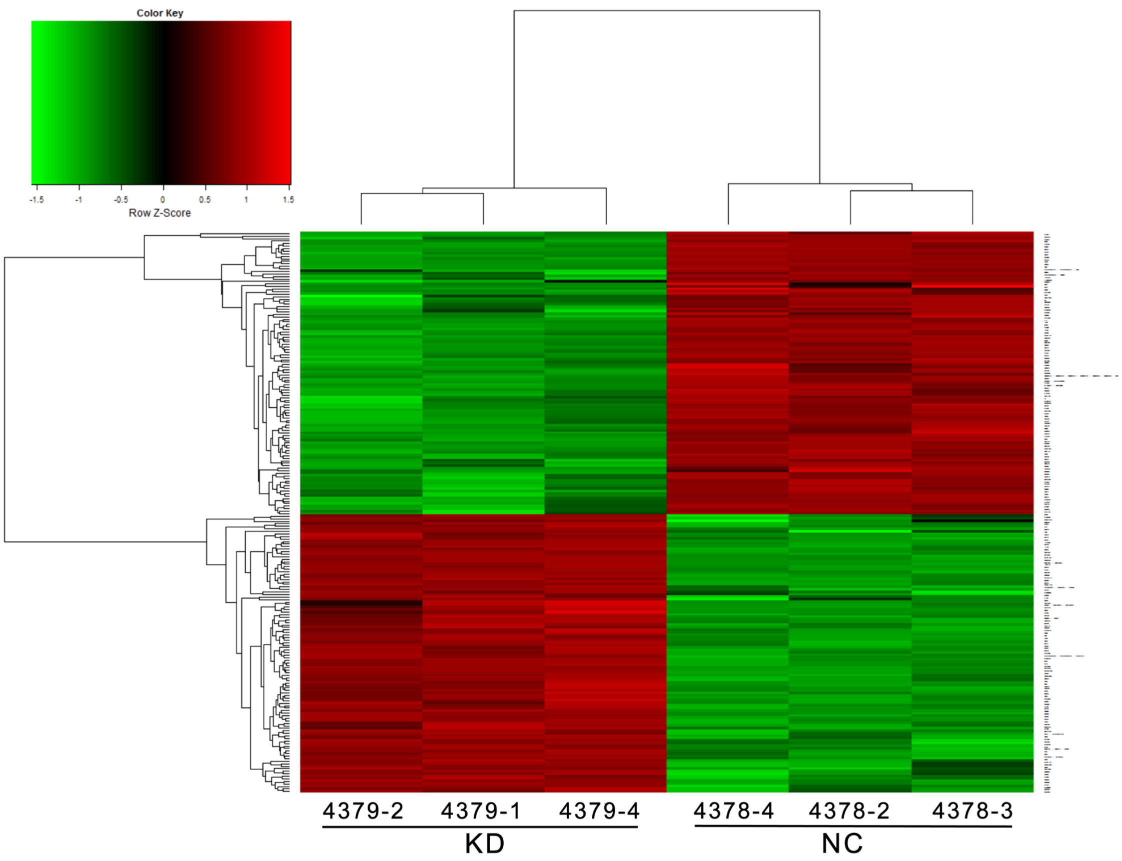

B

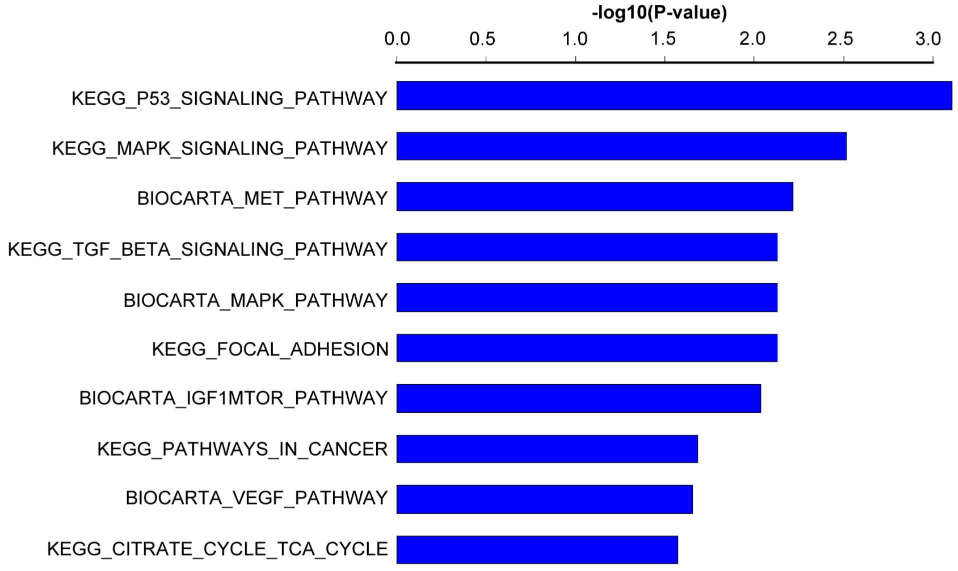

Supplementary Figure 2

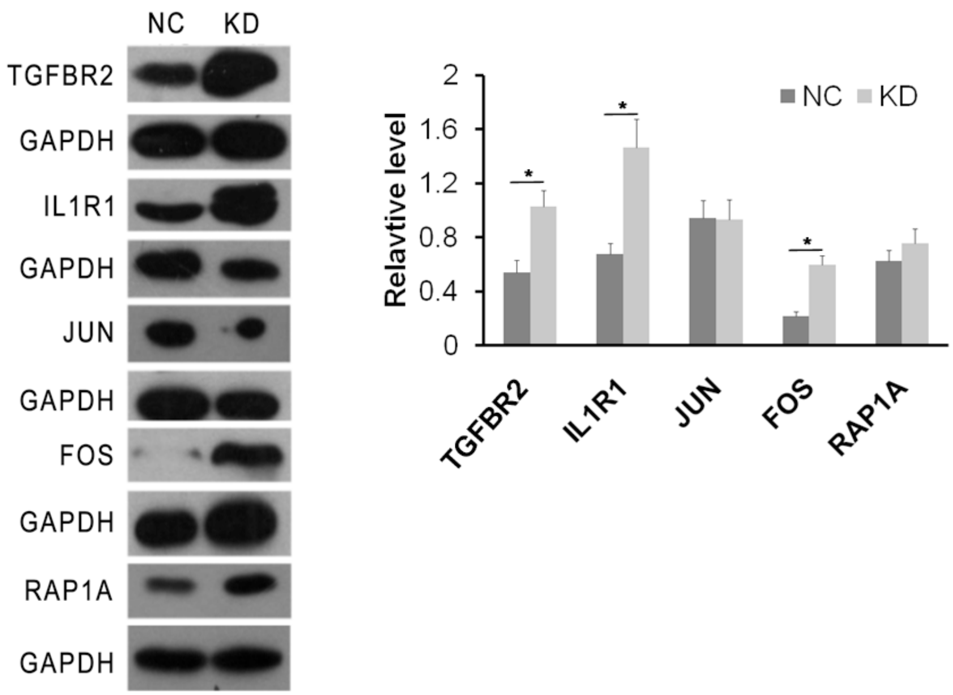

Supplementary Figure 3
